# COVID-19 and Sick Leave: An Analysis of the Ibermutua Cohort of Over 1,651,305 Spanish Workers in the First Trimester of 2020

**DOI:** 10.3389/fpubh.2020.580546

**Published:** 2020-10-19

**Authors:** Eva Calvo-Bonacho, Carlos Catalina-Romero, Carlos Fernández-Labandera, Ana Fernández-Meseguer, Arturo González-Quintela, Paloma Martínez-Muñoz, Luis Quevedo, Pedro Valdivielso, Miguel Ángel Sánchez-Chaparro

**Affiliations:** ^1^Departamento de Proyectos Sanitarios, Ibermutua, Mutua Colaboradora con la Seguridad Social 274, Madrid, Spain; ^2^Departamento de Medicina Interna, Hospital Universitario, Universidad de Santiago de Compostela, A Coruña, Spain; ^3^Servicio de Medicina Interna, Hospital Virgen de la Vic, Málaga, Spain; ^4^Departamento de Medicina y Dermatología, Universidad de Málaga and Instituto de Investigaciones Biomédicas de Málaga (IBIMA), Málaga, Spain

**Keywords:** SARS-CoV2, COVID-19, sick leave, costs, Spain

## Abstract

**Objectives:** The worldwide SARS-COV2 pandemic has impacted the health of workers and companies. The aim is to quantify it according to sick leave.

**Methods:** Using ICD-9 codes, we analyzed Ibermutua records of all sick leaves during the first trimester of 2020, compared to during the same months of 2017, 2018, and 2019. We stratified the analysis by causes, patient sex, activity sectors, and regional data. All sick leaves were adjusted by the number of Ibermutua-affiliated persons in each period.

**Results:** In March 2020, there was an unprecedented (116%) increase in total sick leaves, mainly due to infectious and respiratory diseases. Men and women were equally affected. All activity sectors were impacted, with the highest increase (457%) observed among health-related workers, especially due to contagious disease. The incidences of sick leaves were heterogeneous among different regions. Cost-analysis of sick leaves during the first trimester of 2020 compared with in previous years showed 40.3% increment (mean 2,813 vs. 2,005 € per 100 affiliated workers).

**Conclusions:** The SARS-COV2 pandemic is having a huge impact on workers' health, as shown by data regarding sick leaves in March 2020. This is associated with greater economic burden for companies, both due to the cost associated with sick leaves and the losses in productivity due to confinement.

## Introduction

Since December 2019, starting in the city of Wuhan, China, the world has been suffering an outbreak of the new coronavirus SARS-COV2, which induces acute respiratory infection, bilateral pneumonia, respiratory failure, and in some cases death (1). The rapid dissemination of this virus has forced many national authorities to restrict individuals' movements and to close industries that are considered non-essential, including tourism companies and air travel. In Spain, such measures have been mandatory since March 14, 2020 (2).

The first two cases identified in Spain were imported from Germany (January 31, 2020) and the UK (February 10, 2020). These cases were followed by a sharp rise in the number of infected individuals. At the end of March 2020, there were 85,195 known cases (3), and the mortality was 34% higher than expected (4). This rapid spread overwhelmed many health facilities, necessitating the construction of auxiliary sanitary centers. In the acute setting, COVID-19 diagnosis relies on PCR-based viral detection from nasopharyngeal swabs. However, access to these kits has been limited and some infected individuals remain asymptomatic or with only mild symptoms (5); thus, it is generally agreed that the disease is markedly underdiagnosed.

Sick leave (SL) is a complex indicator of the working population's well-being and also a predictor for health consequences and mortality (6, 7). Moreover, if paid sick leave is guaranteed from the 1st day of illness, it encourage workers to stay at home preventing the spread of virus infection (8).

Ibermutua is a mutual insurance company in occupational medicine, which collaborates with the National Public Health System in Spain to provide healthcare for the working population. Ibermutua covers over 1.6 million workers, and has nearly 100 of its own health centers and 1,000 external health centers, spread throughout Spain. Regarding non-work-related or common diseases (CD), Ibermutua receives daily information from the National Public Health System about its covered workers who are on sick leave, and the cause of it. This enables assessment of the impacts of CD on health in a large sample of workers, and the associated economic burden on society.

The impact of COVID-19 pneumonia on SL and its costs have not been reported in our knowledge, despite of the important component of the economic burden of the disease and the loss of productivity due to morbidity and premature death. In the present study, we aimed to analyze the CD-related SL (CD-SL) of workers during the first trimester of 2020. We compared these data with the findings from previous years, with special focus on health-related workers.

## Methods

### Study Design and Participants

We performed a retrospective comparative analysis that included all CD-SL episodes started between January and March 2020 among workers covered by Ibermutua. We compared these data with the CD-SL episodes during the same months in the years 2017–2019. Data on these episodes were obtained from the National Public Health System Register for SL due to CD.

On January 31, 2020, Ibermutua covered a total of 1,651,305 workers (55.1% men; 44.9% women). The mean age was 42 years (SD, 11.36; range, 16–80 years) among all workers, 42.2 years among men, and 41.9 years among women. On this date, the Spanish Social Security covered 19,171,039 workers (53.4% men; 46.3% women), which included the workers affiliated with Ibermutua (9) The Ibermutua-affiliated sample is considered to provide adequate representation of the Spanish working population, as previously published (6, 10).

Ibermutua is a mutual insurance company that provides healthcare for work-related SL episodes and occupational illnesses. They are also responsible for the management of CD-SL for some companies. Mutual insurance companies in Spain collaborate with National Social Security to administer statutory sick pay. Among its preventive activities, it launched the ICARIA (Ibermutuamur CArdiovascular RIsk Assessment) project in 2004 (11, 12).

In the present study, workers were classified into four major activity sectors (agriculture, construction, industry, and service) according to the Spanish Classification of Economic Activities. The sample included workers from all activity sectors and occupational categories, with a broad age range, and from both genders. We performed separate analysis of CD-SL in health-related workers.

### Sick Leave Definitions and Cost

SL episodes due to infectious disease, cardiovascular disease, respiratory disease, and undefined symptoms were identified based on the International Classification of Diseases, 9th Revision, Clinical Modification (ICD-9-CM) codes 001–139, 390–459, 460–519, and 780–799, respectively. Additionally, cases of COVID-19 infection were identified using the specific ICD-9-CM codes released on March 6, 2020: non-specified viral infection (codes 079) and exposure to contagious disease (codeV01) (13). SL cost was calculated by multiplying the regulatory base (contribution base of the month prior to the SL divided by 30 days) by the SL duration. To compare SL cost in 2020 with costs in 2017, 2018, and 2019, we adjusted total costs in every trimester by the number of workers affiliated in each period.

### Statistical Analysis

Data analyses were performed using Excel 2016 (Microsoft Suite, United States). Data are shown as absolute values and percentages. For comparison with the SL in the first 3 months of 2020, to balance the year-to-year variability, we used the mean SL corresponding to the same months of the years 2017–2019. Data on common SL were adjusted by the number of Ibermutua-affiliated workers every month, which is shown in Supplementary File. We also performed analyses with stratification by gender, activity sector, and autonomous communities.

### Ethical Issues

The research described herein adhered to the tenets of the Declaration of Helsinki. All medical records were anonymized; only statistical information was provided by Ibermutua for research purposes.

## Results

**Figure 1:**
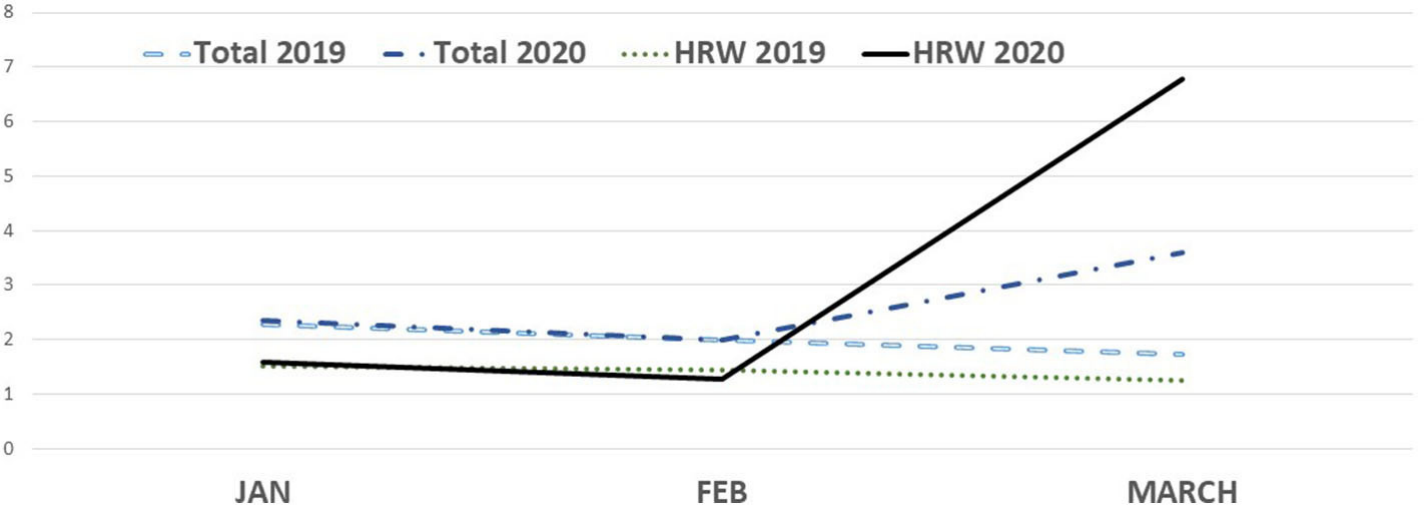
Total and Health-Related Workers (HRW) according to months and years, 2020 vs. 2019. Data are shown adjusted by affiliation.

Table 1 shows total and specific diagnosis of CD-SL during the first trimester of each year from 2017–2020. In 2020, the number of SL in March was increased compared with during the same period in 2017, 2018, and 2019. This increase was especially notable among the codes of infectious disease, undefined symptoms (including malaise, feverishness, myalgia, arthralgia, etc.), and respiratory disease. During the same time period, SL for cardiovascular codes remained stable across all years. We observed a particularly dramatic increase of SL for respiratory diseases in March 2020, accounting for 4.9 cases per 1,000 workers, compared to 2.5 cases per 1,000 workers during this period in 2017, 2018, and 2019. Figure 1 shows changes across the study period. In 2017–2019, SL decreased with progression through the first trimester, and this expected decrease was not observed in 2020. SL clearly increased in March, by 96% for respiratory diseases and by 264% for infectious diseases. The most striking finding of our study was the SL for workers exposed to contagious diseases, which was scarcely reported in 2017–2019, while 4,539 such cases were reported in March 2020, 2.9 cases per 1,000 workers (Table 1).

**Table 1:** Total and most relevant diagnosis related sick leaves episodes in general workers and in health-related workers.

	**Total common sick-leaves episodes**						**Total common sick-leaves episodes in health -related workers**					
	**JAN**		**FEB**		**MARCH**		**JAN**		**FEB**		**MARCH**	
	***N***	**% W**	***N***	**% W**	***N***	**% W**	***N***	**% W**	***N***	**% W**	***N***	**% W**
**Total**												
2017	26,689	2.01	21,310	1.61	24,420	1.80	1,953	1.78	1,506	1.37	1,506	1.37
2018	32,772	2.41	24,412	1.80	20,291	1.47	1,928	1.73	1,426	1.28	1,207	1.06
2019	36,045	2.28	31,291	1.98	27,785	1.72	1,982	1.52	1,878	1.43	1,666	1.25
2020	38,921	2.36	32,785	1.99	57,236	3.60	2,132	1.58	1,739	1.27	9,289	6.78
**Infectious**												
2017	2,083	0.16	1,752	0.13	1,919	0.14	201	0.18	162	0.15	172	0.16
2018	2,555	0.19	1,959	0.14	1,854	0.13	149	0.13	99	0.09	108	0.10
2019	2,667	0.17	2,077	0.13	2,048	0.13	152	0.12	140	0.11	140	0.11
2020	2,869	0.17	2,164	0.13	8,093	0.51	142	0.11	132	0.10	1,625	1.19
**Cardiovascular**												
2017	588	0.04	502	0.04	575	0.04	30	0.03	15	0.01	25	0.02
2018	580	0.04	559	0.04	428	0.03	24	0.02	16	0.01	23	0.02
2019	633	0.04	672	0.04	658	0.04	22	0.02	31	0.02	30	0.02
2020	730	0.04	686	0.04	590	0.04	34	0.03	26	0.02	31	0.02
**Respiratory**												
2017	8,900	0.67	4,110	0.31	3,223	0.24	668	0.61	245	0.34	186	0.17
2018	12,824	0.94	6,590	0.48	3,780	0.27	707	0.63	378	0.44	232	0.20
2019	11,257	0.71	8,108	0.51	4,020	0.25	619	0.48	492	0.31	243	0.18
2020	11,295	0.68	7,517	0.45	7,858	0.49	634	0.47	406	0.30	633	0.46
**Undefined Symptoms**												
2017	2,186	0.16	2,163	0.16	2,621	0.19	30	0.03	15	0.01	25	0.02
2018	2,685	0.20	2,282	0.17	2,096	0.15	24	0.02	16	0.01	23	0.02
2019	4,933	0.31	4,814	0.30	4,929	0.31	22	0.02	31	0.02	30	0.02
2020	6,888	0.42	5,898	0.35	7,874	0.49	200	0.03	157	0.02	341	0.25
**Exposure to Contagious Disease**												
2017	7		0		0		189	0.17	172	0.16	191	0.17
2018	2		2		0		169	0.15	130	0.12	130	0.11
2019	2		4		2		251	0.19	265	0.2	295	0.22
2020	0		0		4,539	0,29	0	0	1	0.0	1,574	1.15
**Non-specified Viral Infection (079)**												
2017	406	0.03	207	0.02	169	0.01	0	0.00	0	0.00	0	0.00
2018	602	0.04	383	0.03	230	0.02	0	0.00	0	0.00	0	0.00
2019	630	0.04	469	0.03	297	0.02	0	0.00	1	0.00	0	0.00
2020	468	0.03	302	0.02	3,013	0.19	12	0.00	9	0.00	830	0.61

*(N = number; %W percent of SL adjusted by affiliation)*.

At the end of March 2020, there were 136,977 Ibermutua-affiliated workers in the sanitary and social services (see Supplementary File). Table 1 shows the total and specific causes of SL among these workers. They exhibited increased total SL, especially associated with respiratory disease, infectious disease, undefined symptoms, and non-specified viral infections. The code associated with SARS-COV2 infection (V01.8 coronavirus SARS-associated) was reported for 1,574 workers in March 2020 (see Figure in Supplementary File showing the percentage of SL among health related workers in the first trimester of the years 2017–2020).

Table 2 shows the distribution of SL during the studied period according to gender and economic activity sector (EAS). No differences were observed between men and women. Additionally, the increase of SL in March 2020 was seen in all EAS, and was higher in construction (284%), agriculture (218%), and industry (163%), compared to in services (85%). SL differed among autonomous regions of the country, with the greatest increases observed in the Basque region, Castilla La Mancha, Madrid, and Navarra. In contrast, the island territories (Balearic and Canary) did not show any SL increase (Supplementary File).

**Table 2:** Distribution of common sick leaves episodes by activity sectors and gender by year.

	**Number**	**% W**	**Number**	**% W**	**Number**	**% W**
**Men**						
2017	12,665	2.18	10,193	1.76	10,929	1.86
2018	16,394	2.59	12,285	1.94	10,264	1.60
2019	18,116	2.08	15,795	1.8	14,079	1.59
2020	20,045	2.2	16,760	1.82	28,848	3.30
**Women**						
2017	14,024	2.96	11,117	2.36	11,491	2.38
2018	16,378	3.18	12,127	2.35	10,027	1.91
2019	17,929	2.53	15,496	2.17	13,706	1.89
2020	18,882	2.55	16,018	2.14	28,388	3.96
**Agriculture**						
2017	308	0.49	277	0.46	322	0.53
2018	407	0.65	297	0.52	255	0.43
2019	528	0.61	515	0.61	479	0.55
2020	599	0.67	547	0.61	1,374	1.59
**Industry**						
2017	3,113	2.36	2,522	1.9	2,633	1.98
2018	4,310	3.13	3,027	2.19	2,420	1.76
2019	4,478	2.47	3,894	2.14	3,321	1.81
2020	4,719	2.57	3,818	2.07	8,613	4.87
**Construction**						
2017	882	1.3	703	1.02	762	1.08
2018	1,240	1.67	872	1.16	746	0.99
2019	1,433	1.19	1299	1.06	1,105	0.88
2020	1,565	1.23	1,350	1.04	4,367	3.77
**Services**						
2017	22,386	2.60	17,808	2.06	18,703	2.15
2018	26,815	3.07	20,216	2.3	16,870	1.89
2019	29,606	2.49	25,583	2.13	22,880	1.88
2020	32,044	2.56	27,063	2.14	44,299	3.65

*N = number; % W: percentage of sick leaves adjusted by affiliation*.

**Figure 2:**
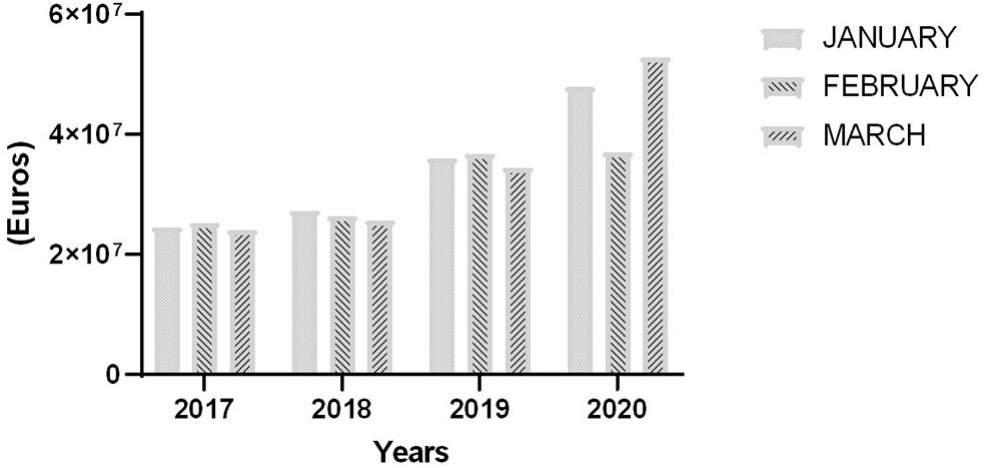
Cost (€) associated to SL, adjusted by affiliation in 2020 compared with the precedent trimesters.

Finally, the increased SL translated into an 40.3% increase of associated costs during the first trimester of 2020 compared with the same period of 2017–2019 (mean 2,813 vs. 2,005 € per 100 affiliated workers). Figure 2 shows the increased cost of SL per 100 workers between January and March 2020 with respect to the same months of the years 2017–2019.

## Discussion

The findings of our present study confirm that in March 2020, there was an unprecedented increase in the number of SL compare with in previous years with the greatest impact on exposed workers (14). The increased SL were mainly due to SL related to respiratory disease, infectious disease, and undefined symptoms, which is concordant with the SARS-COV2 outbreak. Considering the epidemiological context, our results suggest that most of these SL might be related to mild or moderate symptomatic cases of COVID-19. In this context, it is interesting to note that during March 2020, general practitioners in Spain cared for 26 subjects with COVID-19-like symptoms in their clinics for every two patients diagnosed with severe forms of COVID-19 and admitted to hospitals (15). There was no increase of SL in February 2020, even though the first Spanish case of COVID-19 death was identified post-mortem in a patient who died in February, and phylogenetic data indicates that SARS-COV2 was already present in Spain at that time (16).

Increasing clinical and epidemiological evidence suggests that SARS-COV2 infection can induce cardiovascular complications that are associated with a worse prognosis (17, 18). However, we did not find an increase in SL related to cardiovascular disease during 2020. It is possible that cardiovascular diseases associated with COVID-19 might be linked with the severe forms of the disease during evolution, and are rarely the cause of SL.

Concerning economic activity sectors, most SL (in absolute terms) affected the service sector. However, when data were adjusted by affiliated workers to each sector, the SL increase was lowest in the service sector (85%), and the highest increase was observed in the construction sector (284%). Importantly, this finding indicates that no activity sector was safe. It is interesting to note that many of the SL, especially in March 2020, were among health-related workers. Of the 136,977 health-related workers in our study cohort, 6.78% had a SL, compared to 1.22% in previous years. Additionally, 1,574 SL among health-related workers in March 2020 were coded as due to exposure to biological agents. This code has been very rarely used in past years. These results are in agreement with data on confirmed cases of COVID-19 in Spain, where nurses and doctors comprise almost 20% of affected people up to March 2020 (3). In that line, a recent publication in USA found a significant increase in absenteeism in workers in personal care and service groups, including those giving healthcare support during April 2020 (19).

In terms of occupational health, the present situation is a very important issue and a challenge for companies (20), as they should adapt the production of goods to keep their workers at the lowest risk possible, and following the recommendations of the WHO (21). Additionally, regional and national authorities should provide workers with the personal protection equipment required to avoid contact and the widespread transmission of infection at work. This is especially relevant to health-related workers at hospitals and primary care centers, as well as at socio-sanitary institutions (elderly care facilities, etc.). The capacity of health-related workers to face the SARS-COV2 pandemic in Spain has already been recognized (22).

Based on the increased total number of SL in the first trimester of 2020 compared with in previous years, the total cost of SL has also increased by 40.3% reaching 137,722,056 €. This data underestimates the actual cost, since it does not include additional costs faced by workers and companies. Since the Spanish Government decreed confinement starting from March 14, 2020 (2), many non-essential industries have been closed, up to 800,000 workers have become newly unemployed (23), and many other have been affected by temporal regulation of their contracts (furlough). This adds to the fact that over the last 5 years, SL has substantial increased in terms of prevalence and duration, with corresponding increases of associated healthcare costs and productivity losses.

We found that SL were heterogeneous between regional areas, being lower in the Balearic and Canary islands (despite having more tourism activities) and Catalonia, and higher in the Basque region, Madrid, and Castilla-La Mancha. In some instances, our data showed a mismatch with reports of illness in the general population. For instance, Catalonia had the second highest number of patients affected by COVID-19 pneumonia (3), but showed a very small increase in SL. This discrepancy might be related to differences in activity sectors or in the mean age of the population. It is well-known that severe COVID-19 pneumonia predominantly affects elderly people, who are not often affiliated with Ibermutua. Additionally, SL may be affected by the mobility of workers between close regions. This might the case for Castilla-La Mancha and Castilla León, which are very close to Madrid, the largest focal point of COVID-19, with thousands of workers moving between regions every day on high-speed trains. Similarly, Cantabria is close to the Basque region.

One strength of this analysis of non-work-related (common) diseases, is that Ibermutua receives daily official reports from the National Public Health System of Spain about its covered workers who are on a SL and the cause of it. This encoded information (ICD-9–CM) becomes part of the Ibermutua official records. These data were anonymized and used for the present analyses.

As a limitation of this study, it should be noted that during the months of January and February and mid-March, primary care physicians did not have an official regulation for the codification of SL related to the COVID-19 pandemic. This came into use in early March. Thus, especially prior to March, many cases of this infection would have been encoded as undefined symptoms, viral infections, or respiratory infections.

In conclusion, in just the first trimester of 2020, the outbreak of SARS-COV2 has had a dramatic impact on the health of the Spanish working population and on their companies. This influence has been especially severe for health-related workers. Moreover, the impact on health is associated with a significant economic burden on society.

## Data Availability Statement

The raw data supporting the conclusions of this article will be made available by the authors, without undue reservation.

## Ethics Statement

Ethical review and approval was not required for the study on human participants in accordance with the local legislation and institutional requirements. Written informed consent for participation was not required for this study in accordance with the national legislation and the institutional requirements.

## Author Contributions

EC-B, MS-C, and PV made the study design and wrote the draft. CC-R, CF-L, and LQ performed all database analyses and performed data collection. AF-M, PM-M, and AG-Q review all the data and make substantial contribution to data interpretation. All authors interpreted the results, contributed to writing the article, and approved the final version for submission.

## Conflict of Interest

The authors declare that the research was conducted in the absence of any commercial or financial relationships that could be construed as a potential conflict of interest.
