# Supplementary material for: COVID-19 and Sick Leave: An Analysis of the Ibermutua Cohort of Over 1,651,305 Spanish Workers in the First Trimester of 2020
Source: Front Public Health. 2020 Oct 19;8:580546. doi: 10.3389/fpubh.2020.580546 (PMC7604328; doi:10.3389/fpubh.2020.580546)

Supplementary Material

# Supplementary Figures and Tables

**Table Supplementary. Total affiliation, Health-related affiliation and total cost (€) by year and month**.

| **Total affiliation** | January | February | March |
| --- | --- | --- | --- |
| **2017** | 1,327,503 | 1,335,307 | 1,356,042 |
| **2018** | 1,359,699 | 1,359,699 | 1,376,677 |
| **2019** | 1,579,225 | 1,590,265 | 1,610,845 |
| **2020** | 1,651,305 | 1,668,887 | 1,590,920 |
| **Health-related affiliation** |  |  |  |
| **2017** | 109,998 | 109,963 | 109,922 |
| **2018** | 111,592 | 111,591 | 113,340 |
| **2019** | 130,152 | 131,607 | 132,950 |
| **2020** | 135,101 | 136,455 | 136,977 |
| **Total Cost (€)** |  |  |  |
| **2017** | 24,484,779 | 25,276,986 | 24,003,041 |
| **2018** | 27,268,234 | 26,349,558 | 25,657,552 |
| **2019** | 35,953,525 | 36,783,802 | 34,414,854 |
| **2020** | 47,879,722 | 37,011,699 | 52,830,635 |
|  |  |  |  |

**Table 2**

**Percentage of sick leaves in Community autonomous adjusted by affiliation**

|  | **JAN** | **FEB** | **MARCH** |
| --- | --- | --- | --- |
|  | % W | % W | % W |
| **Andalucía** |  |  |  |
| **2017** | 1.99 | 1.7 | 1.88 |
| **2018** | 2.66 | 1.91 | 1.58 |
| **2019** | 2.02 | 1.81 | 1.55 |
| **2020** | 2.37 | 1.92 | 2.86 |
| **Aragón** |  |  |  |
| **2017** | 4.13 | 1.96 | 1.98 |
| **2018** | 4.05 | 3.13 | 2.54 |
| **2019** | 3.56 | 3.75 | 3.04 |
| **2020** | 4.3 | 3.13 | 5.02 |
| **Canary Island** |  |  |  |
| **2017** | 2.64 | 1.88 | 2.68 |
| **2018** | 3.12 | 2.54 | 1.93 |
| **2019** | 2.7 | 2.34 | 2.22 |
| **2020** | 2.72 | 2.42 | 2.57 |
| **Cantabria** |  |  |  |
| **2017** | 2.87 | 1.88 | 2 |
| **2018** | 2.64 | 2.16 | 2.16 |
| **2019** | 3.53 | 1.93 | 2.02 |
| **2020** | 3.47 | 2.73 | 4.65 |
| **C, La Mancha** |  |  |  |
| **2017** | 2.77 | 2.27 | 2.3 |
| **2018** | 3.17 | 2.5 | 2.09 |
| **2019** | 2.8 | 2.52 | 2.08 |
| **2020** | 2.52 | 2.25 | 9.2 |
| **C, León** |  |  |  |
| **2017** | 2.13 | 1.6 | 1.75 |
| **2018** | 2.28 | 1.9 | 1.65 |
| **2019** | 2.42 | 2.11 | 1.82 |
| **2020** | 2.41 | 1.89 | 2.06 |
| **Catalonia** |  |  |  |
| **2017** | 5.73 | 4.26 | 4.72 |
| **2018** | 6.45 | 4.83 | 4.13 |
| **2019** | 6.1 | 5.05 | 4.33 |
| **2020** | 5.71 | 4.97 | 5.65 |
| **Madrid** |  |  |  |
| **2017** | 3.27 | 2.5 | 2.51 |
| **2018** | 3.35 | 2.71 | 2.24 |
| **2019** | 3.42 | 2.91 | 2.58 |
| **2020** | 3.56 | 2.99 | 5.95 |
| **Navarra** |  |  |  |
| **2017** | 5.97 | 3.73 | 3.34 |
| **2018** | 6.19 | 4.77 | 4.26 |
| **2019** | 6.37 | 4.83 | 3.18 |
| **2020** | 5.4 | 3.55 | 6.47 |
| **Valencia** |  |  |  |
| **2017** | 1.74 | 1.42 | 1.4 |
| **2018** | 2.27 | 1.62 | 1.26 |
| **2019** | 1.67 | 1.55 | 1.22 |
| **2020** | 1.51 | 1.4 | 2.89 |
| **Extremadura** |  |  |  |
| **2017** | 1.82 | 1.3 | 1.43 |
| **2018** | 1.7 | 1.61 | 1.28 |
| **2019** | 1.63 | 1.72 | 1.44 |
| **2020** | 1.59 | 1.38 | 2.83 |
| **Galicia** |  |  |  |
| **2017** | 2.36 | 1.82 | 1.89 |
| **2018** | 3.04 | 2.42 | 1.78 |
| **2019** | 1.26 | 1.18 | 0.99 |
| **2020** | 1.58 | 1.25 | 1.92 |
| **Balearic Island** |  |  |  |
| **2017** | 3.5 | 2.33 | 2.77 |
| **2018** | 4.61 | 3.39 | 2.63 |
| **2019** | 3.56 | 2.71 | 2.58 |
| **2020** | 3.13 | 2.5 | 3.17 |
| **La Rioja** |  |  |  |
| **2017** | 3.06 | 1.81 | 2.7 |
| **2018** | 3.76 | 2.65 | 2.49 |
| **2019** | 3.46 | 2.48 | 2.57 |
| **2020** | 2.95 | 3.05 | 6 |
| **Basque Country** |  |  |  |
| **2017** | 4.78 | 2.69 | 2.93 |
| **2018** | 4.55 | 2.84 | 2.8 |
| **2019** | 5.18 | 2.73 | 3.03 |
| **2020** | 4.21 | 3.53 | 7.03 |
| **Asturias** |  |  |  |
| **2017** | 1.17 | 0.85 | 0.99 |
| **2018** | 1.37 | 0.93 | 0.87 |
| **2019** | 1.25 | 1.06 | 0.94 |
| **2020** | 1.68 | 0.91 | 3.09 |
| **Murcia** |  |  |  |
| **2017** | 1.88 | 1.75 | 1.78 |
| **2018** | 2.98 | 1.93 | 1.5 |
| **2019** | 2.16 | 1.75 | 1.56 |
| **2020** | 2.03 | 1.87 | 3.45 |

## Supplementary Figures

Figure Legend: Total SL among HRW, adjusted by the number of Ibermutua-affiliated persons in each period.


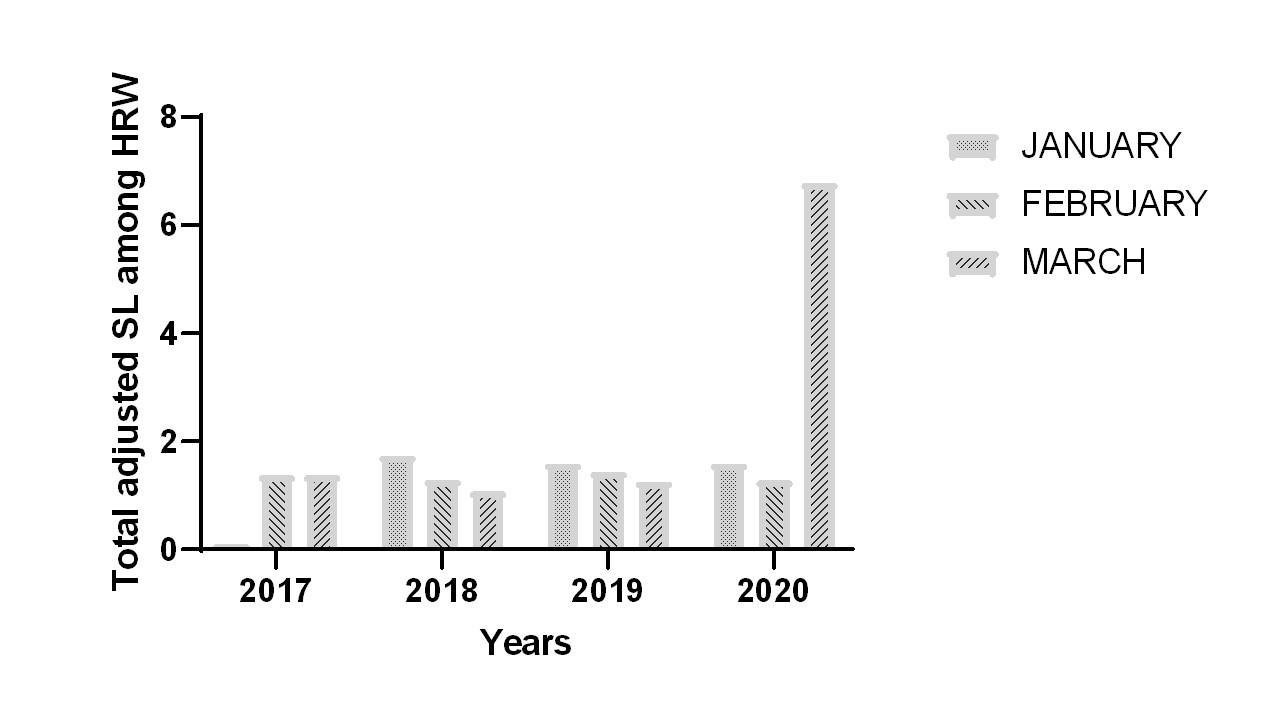

Supplement: Supplementary file 1 [file Table_1.docx]
